# Evaluation of the treatment of equine glandular gastric disease with either long‐acting‐injectable or oral omeprazole

**DOI:** 10.1002/vms3.728

**Published:** 2022-02-15

**Authors:** Sarah Gough, Gayle Hallowell, David Rendle

**Affiliations:** ^1^ Rainbow Equine Hospital Malton North Yorkshire YO17 6SG; ^2^ School of Veterinary Medicine and Science University of Nottingham Nottingham United Kingdom

**Keywords:** gastrointestinal, horse, proton‐pump inhibitor, stomach

## Abstract

**Background:**

Equine glandular gastric disease (EGGD) is common in domesticated horses and can be challenging to treat. Oral omeprazole (ORLO) is used widely but the clinical response is frequently poor.

**Objectives:**

To compare rates of EGGD healing and improvement between ORLO and a long‐acting injectable omeprazole preparation (LAIO).

**Study design:**

Retrospective clinical study.

**Methods:**

The case records and gastroscopy images of horses presenting to *masked for peer review* over a 12‐month period were reviewed, with images blindly assessed by one of the authors. Treatment responses to 4 mg/kg LAIO administered every 7 days for 2 and 4 weeks were compared with ORLO 4 mg/kg PO q24hrs for 4 weeks. Data were compared using a Mann‐Whitney U test with post‐hoc Dunn's test, Chi‐squared test and a Fisher's exact test.

**Results:**

Thirty‐three horses that received LAIO and 12 that received ORLO were identified. Nine horses in the LAIO had received other treatments previously. The groups were comparable in signalment and EGGD lesion severity. Long‐acting injectable omeprazole was found to be non‐inferior to ORLO. LAIO was associated with better healing rates than ORLO at 4 weeks (LAIO‐80%; ORLO‐42%; p = 0.02), and reduction in lesion severity at 2 and 4 weeks in the LAIO group but not in the ORLO group at 4 weeks. Eighteen percent of horses in the LAIO group and 50% in the ORLO group did not heal at 4 weeks. There was no association between rate of healing or improvement and resolution or improvement of clinical signs. Six localised and self‐limiting injection site reactions were identified in 4 horses treated with LAIO (6.7%).

**Main limitations:**

Retrospective design, small numbers and the use of other treatments prior to use of LAIO.

**Conclusions:**

LAIO was found to be non‐inferior to oral omeprazole for EGGD. Larger blinded randomised clinical trials are justified.

## Abstract 2

Equine glandular gastric disease (EGGD) is common in domesticated horses and can be challenging to treat. Long‐acting injectable omeprazole was found to be non‐inferior to ORLO. LAIO was associated with better healing rates than ORLO at 4 weeks (LAIO‐80%; ORLO‐42%; p = 0.02), and reduction in lesion severity at 2 and 4 weeks in the LAIO group but not in the ORLO group at 4 weeks.

## 1 INTRODUCTION

Equine glandular gastric disease (EGGD) is associated with glandular gastritis (Rendle et al., 2018; Crumpton et al., 2015). It has a high prevalence in all populations of horses, although the prevalence is generally higher in horses that perform greater amounts or higher intensity exercise (Sykes et al., 2015). The pathogenesis of EGGD is ill‐defined and poorly understood. In physiologically normal conditions the gastric contents is acidic with a pH of approximately 1–3, and as such, in contrast to equine squamous gastric disease (ESGD), EGGD is thought to result from the breakdown of the normal mucosal defence mechanisms rather than primary acid injury (Sykes et al., 2015; Rendle et al., 2018). Within the antrum of the stomach there are no oxyntic glands and instead mucous and mucin‐secreting cells predominate, resulting in production of a bicarbonate ion‐rich mucous layer that enables development of a pH gradient returning the pH to near neutral on the mucosal surface (Murray, 1992; White et al., 2009). Additionally mucosal blood flow aids in gastro‐protection, and both blood flow and secretion of the mucous barrier are mediated by tightly regulated prostaglandin release (Morrissey et al., 2008).

Oral omeprazole (ORLO) was authorised for the treatment and prevention of recurrence of gastric “ulcers” in the United States in 1999 and shortly thereafter in other countries and has been used for the treatment of EGGD. Authorisation was granted based on its efficacy for the treatment of squamous ulceration; efficacy in treating glandular lesions was not assessed (Andrews et al., 1999; MacAllister et al., 1999), since there was a rudimentary understanding of the disease and lack of availability of endoscopes of sufficient length (Murray et al., 2001). More recent work has indicated poor healing (9‐21%) of EGGD when ORLO is used alone (Sykes et al., 2015; Sykes et al., 2014). The poor response rate of EGGD following treatment with ORLO have prompted clinicians to investigate unauthorised alternative treatments such as misoprostol (Varley et al., 2019), sucralfate (Hepburn, 2014) and a long‐acting intramuscular injectable formulation of omeprazole (LAIO) (Sykes et al., 2017). A pilot study has demonstrated improved outcomes (75% healed, 25% improved) in Thoroughbred racehorses being treated for EGGD with LAIO (Sykes et al., 2017).

The aim of the study was to determine the efficacy of LAIO in treating EGGD in a range of competition horses. It was hypothesised that LAIO would be non‐inferior to ORLO for EGGD healing.

## 2 MATERIALS AND METHODS

### 2.1 Horses

Horses were identified by searching clinical records at *masked for peer review* for horses undergoing gastroscopy between May 2017 and May 2018. Cases were excluded if clinical records or gastroscopy images before and after treatment were incomplete or if additional pharmacological agents were administered concurrently. The occurrence of adverse events was based on clinical signs recorded in clinical records at the time of repeated injection in horses in the LAIO group. Gastroscopy images were anonymised and were then reviewed by one of the authors (*masked for peer review*) who was blinded to the treatment group. Presenting signs, prior treatment for EGGD, improvement in clinical signs and improvement of lesions were evaluated. Treatments were chosen based on clinician and owner preference with informed consent. Feeding and management instructions included increasing pasture turn‐out and days without exercise, increasing access to forage and reducing cereal based rations with the addition of vegetable oil at 0.5‐1ml/kg bwt (unless body condition score was above 4/5 (Carroll & Huntington, 1988)). ORLO administration was recommended after overnight fasting.

Horses in the LAIO group received 4 mg/kg bwt IM of a 100mg/ml omeprazole formulation^1^ into the gluteal muscles on days 0 and 7. Gastroscopy was repeated after 2 injections (around 14 days) and, if lesions had not resolved, again at around 28 days after 2 further injections at 7‐day intervals. Horses in the ORLO group were given 4 mg/kg of a licensed oral omeprazole paste^2^, ^3^ q24hrs PO for around 28 days after which gastroscopy was repeated. Horses in the ORLO group did not undergo gastroscopy at 14 days.

### 2.2 Gastroscopy

Gastroscopy was performed using a 3m flexible videoendoscope.^4^, ^5^ The mucosa of the pyloric antrum was visualised in all horses and a variable portion of the glandular mucosa of the body of the stomach was visible due to a small amount of fluid or residual feed in all horses. Lesions were described in line with recent recommendations (Sykes et al., 2015) by severity (mild, moderate or severe), by lesion contour (depressed, flat, raised, nodular) and lesion appearance (erythematous, haemorrhagic, fibrinosuppurative). Lesion severity was transformed into a numerical scale (0‐3) with 0 designated to horses with no lesions noted, 1 designated to mild disease, 2 moderate disease and 3 severe disease. Following treatment, healing was defined as complete resolution, improvement as a subjective lessening of lesion severity, type or appearance or worsened. Clinical signs were recorded at the time of each gastroscopy based on discussions with the owner and case and case records were subsequently reviewed. The squamous mucosa was also examined and all horses with glandular lesions had concurrent ESGD, however the rate of healing for ESGD has been reported separately.

### 2.3 Statistical Analysis

Clinical data were recorded in Microsoft Excel.^6^ Data are presented as median and inter‐quartile ranges (IQR) for continuous data when non‐normally distributed. Odds ratios (OR) and 95% confidence intervals (95% CI) are displayed for binomial data. Data for age and time between gastroscopic examinations was assessed for normality using a Shapiro‐Wilk test and were both non‐normally distributed thus were evaluated using a Mann‐Whitney U test and post‐hoc Dunn's test. Sex, breed, horse use, presenting signs, lesion severity (mild, moderate and severe) and lesion types between the two groups were compared using Chi‐squared test. Either a Chi‐square (if >80% of the groups have a frequency of 5 or greater) or a Fisher's exact test (when <80% of the groups have a frequency of 5 or greater) were used to evaluate healing, improvement and worsening of lesions between groups and associations with resolution of clinical signs. Horses that had lesion healing were also classified as having lesion improvement. Data from horses that had healed after 14 days of LAIO were combined with data from horses examined at 28 days to determine the outcome at the end of treatment with LAIO.

Non‐inferiority statistics were performed to compare the two treatments with a significant difference between groups being 20% or more regarding lesion healing and improvement. An a priori margin of 20% is commonly used when studies of this nature have not previously been published in the literature (Allen & Seaman, 2007) and was used for studying different doses of omeprazole in the horse (Sykes et al., 2015). A recent similar study evaluated a placebo versus misoprostol for the prevention of NSAID‐associated gastro‐intestinal injury in healthy volunteers and the margin was similarly set at 17% (Lee et al., 2011). Two statistical software packages were used.^7^, ^8^ The 95% confidence intervals are displayed using Jeffrey's intervals and were calculated using online statistical software.^9^ Significance was determined when p<0.05.

## 3 RESULTS

### 3.1 Horses

Signalment data of horses are presented in Table 1. 33 horses were treated with LAIO and 12 with ORLO. Of those horses treated with LAIO, 32 were injected in the gluteal muscles, while 1 horse was injected into the pectoral muscles on one occasion. Horses treated with ORLO were treated for a median of 29 days (range 21–38 days). 9 horses being treated with LAIO in the current study period, had received one or more treatments for EGGD previously, but lesions had persisted. These treatments included ORLO (5 horses), esomeprazole (2 horses), sucralfate (2 horses), misoprostol (1 horse) and ranitidine (1 horse).

**TABLE 1:** Signalment data for horses treated with either injectable or oral omeprazole

	Injectable omeprazole	Oral omeprazole	
Number of horses	33	12	
Age	9 (IQR 7–10)	11 (IQR7‐14)	p = 0.25
Mares ‐geldings‐stallions	12‐20‐1	6‐5‐1	p = 0.46
Thoroughbred (pure or cross)	4 (12.1%)	3 (25%)	p = 0.03
Warmbloods	11 (33.3%)	2 (16.7%)	
General riding	16 (458.5%)	3 (258%)	P = 0.02
Showjumping	8 (24.221%)	0 (08%)	P = 0.02
Dressage	4 (12.1%)	3 (25%)	
Hunting and eventingEventing	3 (9.133%)	0 (017%)	
RacingOther	2 (36.1%)	6 (508%)	
Days to approximate 28 day re‐examination	28 (IQR = 27.25‐28)	29 (IQR = 24‐33)	p = 0.51
Previous treatment	9 (27.330%)	0%	

### 3.2 Presenting signs and clinical response to treatment

Presenting clinical signs are shown in Table 2. Horses treated with ORLO demonstrated a greater number of clinical signs. Overall, 53% of horses demonstrated two or more clinical signs (LAIO‐39.4% and ORLO–100%; p = 0.0003). Girthing pain and poor performance (overall–20%; LAIO–15.1% and ORLO‐33%; p = 0.02) and poor performance and changes in behaviour (overall–17.8%; LAIO–9.1% and ORLO‐41.7%; p = 0.22) were the two most common combinations of clinical signs reported

**TABLE 2:** Clinical signs at presentation for horses treated with injectable or oral omeprazole

	Total horses	Injectable omeprazole	Oral omeprazole
Poor performance	22 (48.9%)	14 (42.4%)	8 (66.7%)
Behaviour change	15 (33.3%)	8 (24.2%)	7 (58.3%)
Girthing pain	14 (31.1%)	8 (24.2%)	6 (50%)
Abdominal pain	13 (28.9%)	7 (21.2%)	6 (50%)
Weight loss/poor weight gain	10 (22.2%)	5 (15.2%)	5 (41.7%)
Change in appetite	11 (24.4%)	4 (12.1%)	7 (58.3%)

In 78% of horses there was resolution of clinical signs (91% LAIO, 50% ORLO) at 28 days. However, resolution of clinical signs was not associated with lesion improvement (p = 0.09; OR = 5 (0.9‐21)) or healing (p = 0.65; OR = 1.3 (0.4‐4.5)) in either group at any time point. In 91% of horses there was an improvement in clinical signs (91% LAIO, 92% ORLO) at 28 days. Additionally, improvement in clinical signs was not associated with lesion improvement (p = 0.10; OR = 4.8 (0.9‐25)) or healing (p>0.99; OR = 1.27 (0.3‐5.5)) in either group at any time point.

### 3.3 Gastroscopy findings

All lesions in this study involved the pylorus and/or pyloric antrum and there was no difference in lesion severity (p = 0.58) or contour between groups (p>0.96). Lesion severity was mild (LAIO‐25%; ORLO‐27.3%), moderate (LAIO‐58.3%; ORLO‐ 42.4%) and severe (LAIO‐16.7%; ORLO‐ 30.3%). Lesion contour was flat (LAIO‐54.5%; ORLO‐50%), raised (LAIO‐15.1%; ORLO‐25%), nodular (LAIO‐15.1%; ORLO‐16.7%) or depressed (LAIO‐15.1%; ORLO‐16.7%). Lesion appearance was different between groups (p = 0.03) with lesions being erythematous (LAIO‐51.2%; ORLO‐33.3%), haemorrhagic (LAIO‐27.2%; ORLO‐83.3%) or fibrinosuppurative (LAIO‐18.2%; ORLO‐0%). The combination of contour and appearance was highly variable. The most common combinations seen in this study were flat and erythematous (LAIO – 24%; ORLO‐ 25%), nodular and erythematous (LAIO‐12.1%; ORLO‐8.3%) and flat and haemorrhagic (LAIO‐9%; ORLO‐25%). There was no difference between treatment groups for these combined lesion types (p>0.43).

After 14 days of treatment with LAIO, 17 horses (52%) had healed, 29 (88%) had improved, 2 (6%) were unchanged and 2 (6%) had worsened. This is comparable to the response observed following 28 days of ORLO for healing (LAIO‐52%; ORLO‐50%; p = 0.44; OR = 1.68 (0.42‐6.25), improvement (LAIO‐88%; ORLO‐75%; p = 0.36; OR = 2.4 (0.52‐10.3)) and those that either were unchanged or worsened (LAIO‐12%; ORLO‐25%; p = 0.36; OR = 2.4 (0.52‐10.3)).

At the end of treatment (14 or 28 days) with LAIO 27 horses (82%) had healed, 30 horses (91%) had improved and 3 horses (9%) were unchanged or had worsened. Following treatment with ORLO 6 horses (50%) had healed, 9 (75%) had improved and 3 (25%) were unchanged or had worsened. The number of horses that had healed was greater for horses treated with LAIO compared to ORLO (LAIO‐82%; ORLO‐50%; p = 0.03; OR = 2.4 (0.52‐10.3)).

### 3.4 Adverse events

There were localised injection site reactions after six injections in four horses treated with LAIO (6.5% of all injections). In one horse, these occurred after three of the four LAIO injections. With the exception of one horse that had been injected in the pectoral muscles, all reactions resolved within 3 days. Ultimately this also resolved without any treatment. No adverse events were reported with ORLO.

## 4 DISCUSSION

This retrospective study has demonstrated non‐inferiority, that is to say pharmacological efficacy, of this novel injectable treatment (LAIO) when compared with oral omeprazole, for improvement and healing of EGGD when 4 doses were administered but not when 2 doses were administered (Table 3). Notably, 18% of horses in the LAIO group and 50% in the ORLO group did not heal at around 28 days resulting in a large number of non‐responders (Table 3). As this was retrospective, it was not possible to set out with an aim to demonstrate superiority as one might with a prospective, randomised controlled trial or if LAIO was being compared with a placebo product. As such these statistics were not performed. Analysis of these data have shown greater healing and improvement of EGGD lesions when treated with LAIO compared to ORLO for 28 days. The rates of healing following LAIO administration reported here are lower than previously reported in Thoroughbred racehorses (Sykes et al., 2017), although the rates of improvement were similar to the healing rates in that previous study. Furthermore, deterioration of lesions during treatment was not reported previously with LAIO, although has been previously reported with ORLO. The current study also demonstrated that healing and improvement following treatment with LAIO for 14 days was not different to the outcomes following treatment with ORLO for 28 days, and as such the 14 day treatment protocol previously evaluated would not appear relevant in sports and leisure horses (Sykes et al., 2017). The difference in efficacy of LAIO in the current study compared to the study by Sykes et al. (2017) may indicate differences in lesion type or treatment response between a Thoroughbred racing population and a sport and leisure population. Alternatively, as Thoroughbreds and Thoroughbred crosses were the most common type in this study, it may simply reflect the low number of horses evaluated in both studies, or simply a difference in the classification of what constitutes healing given this is a subjective assessment. The relative efficacy of LAIO and ORLO was not evaluated in the previous study evaluating LAIO and therefore comparisons between outcomes may be indicative of differences in lesion severity in the present study. Inclusion of horses in the LAIO group previously that had failed to respond to other treatments (including ORLO) may have impacted on the healing and improvement rates seen with LAIO as they may represent more challenging lesions to treat and explain why these rates were lower than previously published (Sykes et al., 2017). However, rates of healing with ORLO were greater than previously reported (Sykes et al., 2015; Sykes et al., 2014; Varley et al., 2019) and higher than reported when ORLO was combined with sucralfate (Varley et al., 2019; Hepburn & Proudman, 2014).

**TABLE 3:** Non‐inferiority analysis of treatment failures when the control (oral omeprazole) was compared with the novel treatment (injectable omeprazole). The injectable omeprazole treatment for healing but not improvement could be shown to be non‐inferior to oral omeprazole

	Failure rates			
	Oral omeprazole at 28 days	Injectable omeprazole at 28 days	Difference in failure (%): Control treatment failure (omeprazole) minus novel treatment (injectable omeprazole) %	Upper 90% confidence interval
Glandular healing	50% (6/12)	18% (6/33)	32%	39%
Glandular improvement	25% (3/12)	9% (3/33)	16%	24%

There were a variety of lesion types identified at the pyloric antrum and there were more flat, erythematous lesions treated in the ORLO group. We currently do not know which lesions are more severe, whether different lesions have different underlying causes or are likely to respond to different therapies. One abattoir study demonstrated that for many horses with lesions at the pyloric antrum, a generalised gastritis was seen histologically and that pyloric lesion severity did not correlate with the degree of gastritis (Crumpton et al., 2015). These key findings make it extremely challenging in retrospective studies to compare two different pharmacological agents when the underlying natural history of the lesions is unknown. However, this study using clinical cases is more relevant to clinical practice. It is anecdotally reported (Hepburn, personal communication) that flat, haemorrhagic lesions take longer to heal and this might explain some of the differences in healing rates between ORLO and LAIO.

It has been suggested that EGGD lesions typically require 8–12 weeks of oral treatment to heal and even then, many remain refractory to treatment (Sykes et al., 2015). The main clinical sign attributed to gastric disease was poor performance alongside other signs, which is similar to that found in a previous study (Varley et al., 2019).

Regardless of treatment group there was no association between improvement in gastroscopic appearance of the stomach and improvement in clinical signs. This may be due to the non‐specific nature of the clinical conditions with which many horses presented, a perceived placebo effect of the ‘new’ novel treatment or improvements in comfort with increased gastric acidity. It does however highlight the difficulty of assigning clinical significance to gastric lesions (Sykes et al., 2015).

Whilst there is a widely held view that once daily administration of ORLO results in 24 hours of acid suppression based on early studies (Jenkins et al., 1992; Daurio et al., 1999), the average duration of acid suppression with oral treatment at 4 mg/kg was under 12 hours in one study (Merritt et al., 2003) and may be much lower in horses on a high forage diet (Sykes, 2016). In many animals the duration of acid suppression with ORLO may not be sufficient for EGGD healing, particularly in the pyloric region where pH is lowest. The degree and duration of acid suppression required for healing of the glandular mucosa in horses is unknown, but in man maintenance of pH above 3 for > 16 hours is required for healing of lesions of the gastric mucosa (Bell et al., 1992). Pharmacokinetic studies of LAIO have demonstrated that, compared to oral administration of equivalent doses, it suppresses acid more effectively for a longer period of time so greater rates of healing are expected (Sykes et al., 2017; Sykes et al., 2015) and were observed in this study. Although acid suppression is thought to be central to the management of EGGD, one has to consider that acid injury may not be the primary mechanism of disease instigation (Sykes et al., 2015) and factors other than acid suppression and gastric pH may be important in healing of the glandular mucosa. There are an increasing number of publications trying to investigate the natural history and the findings are highly suggestive that EGGD is a syndrome rather than a specific disease (Rendle et al., 2018). These factors might account for the failure of healing of the horses treated with LAIO in the current study. Insufficient duration of acid suppression might also be a factor as treatment of gastric lesions in humans typically necessitates greater than one month of acid suppressant therapy (Scally et al., 2018) and experience of ORLO indicates that some horses which fail to respond to treatment with 28 days of treatment may heal after a more protracted treatment or after changing to treatment with LAIO (B. Sykes, personal communication).

The apparent increase in efficacy may be due to omeprazole administered via a parenteral route overcoming issues of varied and unpredictable bioavailability that have been identified with ORLO (Sykes et al., 2015) or the more consistent concentrations of omeprazole that are attained at the target receptor with LAIO over each 24‐hour period (Sykes et al., 2017) or both effects. Failure to withhold feed overnight prior to treatment can reduce the bioavailability of ORLO by as much as 66% in some horses and may prevent any increase in pH in the glandular portion of the stomach (Jenkins et al., 1992; Bell et al., 1992). It is possible that poor compliance and failure to follow dietary recommendations might have resulted in a reduction in efficacy of ORLO in the current study. The requests that are made of owners around treatment of EGGD in clinical practice that include fasting prior to the administration of ORLO, exercising 30–60 minutes after a small feed (typically 1–2L of chaff or similar roughage), exercising an hour after the administration of omeprazole and feeding a large roughage meal an hour after the administration of omeprazole, are often impractical and even contradictory with respect to fasting, which can limit compliance. Given what is now known about the effects of feeding on the bioavailability of ORLO it would be logical to use LAIO if dietary recommendations cannot be followed appropriately to ensure efficacy of ORLO. However, given that LAIO is not currently a licenced medication, it's use must be in line with the clinicians respective governing authority for prescription of unlicenced medications.

Injectable drug administration is inevitably accompanied by a risk of injection site reactions. The LAIO preparation used in the current study is oil‐based and highly viscous to limit the rate of uptake from muscle. A preparation of this nature might reasonably be expected to increase the risk of idiosyncratic reactions or infection at the injection site. However, in this study the complication rate was low, recognised as localised and self‐limiting swelling or subcutaneous oedema at the injection site. The fact the reaction seen in the pectoral region was worse than those observed when the drug was injected into the gluteal muscles might suggest avoiding the pectoral muscles if at all possible.

This study has a number of limitations. Case numbers were low in both groups, particularly the ORLO group and this likely impacts on the wide confidence intervals. Disappointing responses in EGGD cases to ORLO historically resulted in clinicians involved in the study utilising alternative therapies such as misoprostol and esomeprazole and administering other treatments such as sucralfate with ORLO thereby limiting the number of horses treated with ORLO alone that were available for inclusion in this retrospective study. Inclusion of horses (29% of the group) that had failed to respond to other treatments (including ORLO) in the past may have impacted on the healing and improvement rates seen with LAIO as they may represent more challenging lesions to treat and explain why these rates were lower than previously published (Sykes et al., 2017).

The retrospective nature of this study and hence absence of prospective random allocation to a treatment group limits the study. Despite the absence of random allocation, there was no difference in age or sex between the two groups. There were differences in breed distribution and discipline between the groups with the most notable findings being the over‐representation of Warmbloods and general riding horses in the LAIO group, and pony club activity in the ORLO group. The differences between groups are probably anomalies resulting from the low number of horses in each group. The severity and distribution of lesions was not different between groups, but there was a difference in lesion type. The potential for bias and differences between groups highlights the need for larger, more robust clinical trials of LAIO.

In conclusion, in a population of sports and leisure horses LAIO was found to be non‐inferior to ORLO. The rate of healing of EGGD was higher with LAIO than with ORLO and treatment for a minimum of 28 days was warranted in this population. However, numbers were small and the study was subject to several limitations as studies in clinical cases often are. Larger, blinded, randomised clinical trials of LAIO are warranted as is a better understanding of the natural history of these lesions and the acceptance that EGGD is a syndrome.

## AUTHORSHIP

All authors made substantial contributions to conception and design of the study, acquisition of data or analysis and interpretation of data and all were involved in drafting the article, revising it critically for important intellectual content and finally approving the submitted manuscript.

## AUTHOR CONTRIBUTION

All authors contributed to the preparation of the initial manuscript, drafting of alterations to the manuscript based on peer review and hence preparation and approval of the final manuscript.

## FUNDING

No funding or other incentive was received for performing the study.

## COMPETING INTERESTS

D. Rendle has previously received payment for consultancy services provided to BOVA UK and Luoda pharma and who produce the LAIO and from Boehringer Ingelheim and Norbrook Animal Health who produce oral omeprazole.

## ETHICAL ANIMAL RESEARCH STATEMENT

Informed client consent was obtained from all owners to use an unlicensed product and to publish clinical data. The study was approved by The University of Nottingham Animal Welfare and Ethical Review Body.

### PEER REVIEW

The peer review history for this article is available at https://publons.com/publon/10.1002/vms3.728.

## Data Availability

The data that supports the findings of this study are available on request.

## ENDNOTES

1 Injectable omeprazole. BOVA UK, 7 Gorst Rd, Park Royal, London, NW10 6LA

2 Peptizole. Norbrook Animal Health, Norbrook Laboratories Limited, Carnbane Industrial Estate, Newry, Northern Ireland, BT35 6QQ

3 Gastrogard. Boehringer Ingelheim, Ellesfield Ave, Bracknell, RG12 8YS

4 Pentax B12303. Pentax House, Heron Drive, Langley, Slough, SL3 8PN, United Kingdom

5 Aohua Vet 1335. Aohua Endoscopy Ltd, Shengang Road, Songjiang District, Shanghai, 201612, China

6 Excel. Microsoft Corporation,  Redmond, Washington, United States

7 GraphPad 4.0. Graphpad Software, La Jolla, California, USA

8 SPSS for Windows 15.0. SPSS Inc, Chicago, Illinois, USA

9
http://epitools.ausvet.com.au/content.php?page=CIProportion
